# USP32 deubiquitinase: cellular functions, regulatory mechanisms, and potential as a cancer therapy target

**DOI:** 10.1038/s41420-023-01629-1

**Published:** 2023-09-07

**Authors:** Shuang Li, Yang Song, Kexin Wang, Guoxiang Liu, Xiaolei Dong, Fanghao Yang, Guang Chen, Can Cao, Huhu Zhang, Mengjun Wang, Ya Li, Teng Zeng, Chunyan Liu, Bing Li

**Affiliations:** 1 Department of Genetics and Cell Biology, School of Basic Medicine, Qingdao University, Qingdao, China; 2 Department of Hematology, The Affiliated Hospital of Qingdao University, Qingdao, China

**Keywords:** Cancer therapy, Post-translational modifications

## Abstract

An essential protein regulatory system in cells is the ubiquitin-proteasome pathway. The substrate is modified by the ubiquitin ligase system (E1-E2-E3) in this pathway, which is a dynamic protein bidirectional modification regulation system. Deubiquitinating enzymes (DUBs) are tasked with specifically hydrolyzing ubiquitin molecules from ubiquitin-linked proteins or precursor proteins and inversely regulating protein degradation, which in turn affects protein function. The ubiquitin-specific peptidase 32 (USP32) protein level is associated with cell cycle progression, proliferation, migration, invasion, and other cellular biological processes. It is an important member of the ubiquitin-specific protease family. It is thought that USP32, a unique enzyme that controls the ubiquitin process, is closely linked to the onset and progression of many cancers, including small cell lung cancer, gastric cancer, breast cancer, epithelial ovarian cancer, glioblastoma, gastrointestinal stromal tumor, acute myeloid leukemia, and pancreatic adenocarcinoma. In this review, we focus on the multiple mechanisms of USP32 in various tumor types and show that USP32 controls the stability of many distinct proteins. Therefore, USP32 is a key and promising therapeutic target for tumor therapy, which could provide important new insights and avenues for antitumor drug development. The therapeutic importance of USP32 in cancer treatment remains to be further proven. In conclusion, there are many options for the future direction of USP32 research.

## Facts

- USP32 is involved in a variety of cell biological processes, such as cell cycle, proliferation, invasion, migration, and DNA damage repair.
- USP32 acts as an oncogene in a variety of tumors. Therefore, USP32-specific inhibitors can be developed to study its role in tumors.
- We summarize the structure and biological function of USP32 and discuss the mechanism of USP32 action in a variety of tumors.

## Open Questions

- The expression of USP32 is often out of balance, especially in cancer. Does USP32 have any other functions among many types of tumors?
- How does USP32, a target for many cancer therapies, specifically regulate the content and function of relevant proteins through deubiquitination?
- USP32 has a regulatory effect on a wide range of tumors, so it is possible to provide ideas for treating tumors by modulating USP32 proteins.

## Introduction

**Fig. 1:**
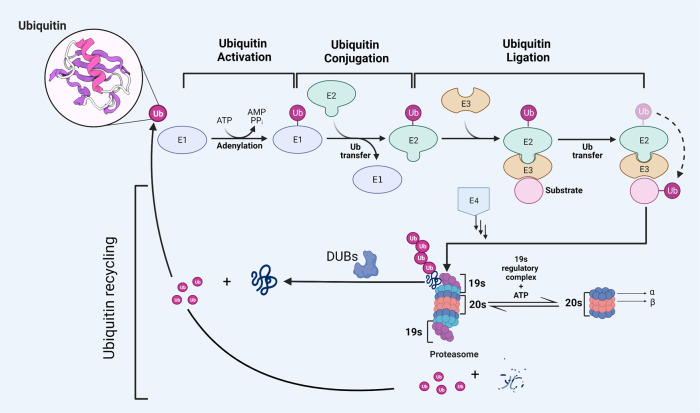
Ubiquitin-proteasome system. Multiple ongoing steps are required for the target protein’s ubiquitin breakdown. Ubiquitin is first activated by E1 enzymes when ATP (adenosine triphosphate) supplies a specific amount of energy. Ubiquitin activase E1 then sends the active ubiquitin molecules to E2 enzymes, and ubiquitin ligase E3 binds E2-binding ubiquitin to the target protein. The substrate protein’s ubiquitin molecule is extended by the E4 ubiquitin chain extension factor, and the tagged protein’s amino acid tail then forms a short ubiquitin molecular chain. Finally, the ubiquitin-tagged substrate protein is selectively recognized by the 26 S proteasome. The binding of 20 s catalytic core particles to 19 s regulatory complex, which binds the substrate protein tagged by ubiquitin chain and transfers the protein substrate to 20 s catalytic core under the energy of ATP, results in the formation of the structure of the 26 S proteasome. The substrate protein is degraded into small oligopeptides of less than 25 amino acids at the proteolytic β subunit of 20 s center, which will eventually be degraded into amino acids by protease in the cytoplasm. Ubiquitin molecules are recovered into the cytoplasmic pool. The deubiquitinating enzymes (DUBs) family hydrolyzes ubiquitin molecules from substrate proteins that include ubiquitin chains by hydrolyzing the ester, peptide, or isopeptide linkages at the carboxyl terminus of ubiquitin, and this process inversely controls protein deterioration. Ubiquitin molecules are also recycled into the cytoplasmic pool to exert their functions.

Proteins are the most important performers of various cellular functions, and their proper function determines whether life activities can be carried out in an orderly and efficient manner, in which post-translational modification (PTM) plays a crucial role [1–3]. As a complex mechanism of biological function regulation, PTM is very important for many key events involving cellular response [4]. In general, intracellular proteins undergo several sorts of modifications upon translation, including phosphorylation, acetylation, methylation, and ubiquitination, each of which is associated with one or more distinct functions [5]. Ubiquitination is one of the post-translational modifications, which refers to the covalent binding of ubiquitin to target proteins catalyzed by a number of different enzymes. The series of enzymes refer to three enzymes that cooperate with each other in the process of ubiquitin cascade: E1 ubiquitin activating enzyme, E2 ubiquitin binding enzyme, and E3 ubiquitin ligase [6, 7]. Hundreds of ubiquitin ligases are found in mammals, suggesting that the diversity of E3 ligases provides an accurate basis for substrate selection [8]. The ubiquitin-proteasome pathway (UPP) is responsible for 80–90% of eukaryotic protein degradation [9]. Ubiquitin is precisely linked to the target protein or to the ubiquitin chain that has already been linked to the target protein under the sequential catalysis of the E1, E2, and E3 enzymes. The E3 ubiquitin ligase controls the target protein’s precise identification, and the human body has an E4 enzyme-ubiquitin chain extension factor that may lengthen the ubiquitin chain to produce a polyubiquitin chain [10] (Fig. 1). In addition, ubiquitin modification can also occur on the N-terminal of protein and some other amino acids (cysteine, serine, threonine) [11, 12].

Ubiquitin is a strictly regulated and reversible process. DUBs can undo ubiquitin modification by hydrolyzing peptide or isopeptide links between ubiquitin molecules or between ubiquitin and substrate proteins [13]. Deubiquitinating enzymes not only inhibit ubiquitin processes, but also promote them by disassembling ubiquitin inhibitors, recycling ubiquitin molecules, and proofreading ubiquitin processes [12], which form a complex regulatory network with the ubiquitin system. Currently, there are seven families of ubiquitin-specific proteases (USPs), ubiquitin c-terminal hydrolases (UCHs), ovarian tumor proteases (OTUs), JAMMs (also known as MPN+), MJDs (also known as Josephins), MINDY family, and ZUP1 family, which make up human DUBs [14]. Cysteine proteases are categorized into six families (USPs, UCHs, OTUs, MJDs, MINDYs, and ZUP1), whereas zinc-dependent metalloproteinases make up the JAMM family [15]. Every family of DUBs is conformed from yeast to humans, with the exception of MJDs [16]. The conversion rate, activation, circulation, and localization of several proteins are all impacted by DUBs activity, which is crucial for intracellular homeostasis, protein stability, and a variety of signal pathways. Alterations in DUB function are simultaneously associated with many diseases, including cancer [17]. It is well known that USPs are the largest class of DUBs, with more than 60 members, and there is growing interest in its function and the important role that substrates play in the pathogenesis of many diseases, including cancer. As was already mentioned, USPs are a class of cysteine-dependent protein hydrolases whose catalytic structural domain (also known as the USP structural domain) is one of their most conserved structural domains. Other domains in USPs include those that can predict ubiquitin binding, such as the zinc finger ubiquitin-specific protease (ZnF-UBP) domain, the ubiquitin interaction motif (UIM) domain, and the ubiquitin associated (UBA) domain [18]. At the same time, although the additional domain around it does not bind to the substrate, the conformational change occurs during ubiquitin binding [18]. Dysfunction in the USPs family can lead to many diseases, including cancer [19], metabolic diseases [20], and neurodegenerative diseases [21], among others. Among them, the research on the relationship between USPs and malignant tumor has become a hot spot, and many studies have shown that targeted intervention of this pathway is expected to become an ideal anti-tumor therapy strategy. We list the function of USPs in carcinogenesis in Table 1.

**Table 1:** Structure of ubiquitin-specific protease (USPs) and its role in cancer progression.

USP	Structure	Cancer of type	Expression of USP	Substrates	Detailed mechanisms	References
USP1		HCC	Upregulated	RPS16	USP1 maintains RPS16 and encourages HCC cell proliferation and metastasis	[74]
		PDAC	Upregulated	BCAT2	After BCAA increases protein synthesis of USP1 through inhibition of the GCN2-eIF2α pathway, USP1 stabilizes BCAT2 protein to promote PDAC	[75]
		PC	Upregulated	KDM4A	USP1 stabilizes KDM4A and promotes PC growth and tumorigenesis	[76]
		NPC	Upregulated	miR-375	MiR-375 regulates USP1, then activates PI3K/Akt pathway, promotes invasion and migration of nasopharyngeal carcinoma cells, and inhibits apoptosis of nasopharyngeal carcinoma cells	[77]
		TNBC	Upregulated	TAZ	USP1 regulates TAZ stability, which favors the development of triple negative breast cancer	[78]
USP2		GBM	Downregulated	SMAD7	By maintaining the stability of SMAD7 and blocking the TGF- signaling pathway, USP2 prevents the growth of GBM	[79]
		GC	Upregulated	E2F4	By stabilizing E2F4, USP2 promotes autophagy and zinc homeostasis and possesses carcinogenicity	[80]
		Lung Cancer	Downregulated	ARID2	By preventing de-ubiquitin ARID2, USP2 prevents lung cancer cells from migrating and invading	[81]
USP3		ESCC	Upregulated	Aurora A	USP3 promotes esophageal squamous cell carcinoma (ESCC) cells to multiply and disseminate invasively through controlling Aurora A	[82]
		GBC	Upregulated	PKLR	USP3 regulates PKLR and affects malignant phenotype and glycolysis of GBC cells	[83]
		NSCLC	Upregulated	RBM4	USP3 controls RBM4 to encourage the growth of NSCLC cells	[84]
USP4		Breast cancer	Upregulated	DNPEP	USP4 is controlled by DNPEP, which is controlled by PAK5 kinase phosphorylation, to advance malignant breast cancer	[85]
		Lung Cancer	Upregulated	Twist1	USP4 promotes the development of lung cancer by stabilizing Twist1 protein	[86]
USP5		Neuroblastoma	Upregulated	MYCN	USP5 drives neuroblastoma by stabilizing MYCN	[87]
		NSCLC	Upregulated	PD-L1	By controlling PD-L1, USP5 aids in the development and incidence of non-small cell lung cancer	[88]
		Breast cancer	Upregulated	HIF2α	USP5 stabilizes HIF2α to promote breast cancer progression	[89]
		GBM	Upregulated	CyclinD1	By controlling CyclinD1 protein stability, USP5 encourages the development and incidence of GBM	[90]
USP6		Ewing Sarcoma	Non	Non	USP6 induces immune response and exerts its inhibitory effect on Ewing’s sarcoma	[91]
USP7		LUAD	Non	Raf-1	By controlling Raf-1 and blocking the ERK1/2 signal pathway, USP7 prevents the growth of lung cancer cells	[92]
		GC	Upregulated	PD-L1	USP7 inhibitors control the stability of p53 to prevent GC cell proliferation and control the expression of PD-L1 to enhance tumor immune response	[93]
		Glioma	Non	XIAP	USP7 promotes glioma development by activating p53-independent pathway to inhibit apoptosis through deubiquitination of XIAP	[94]
USP8		ESCC	Upregulated	ID1	USP8 promotes the development of ESCC tumors by regulating the expression of ID1-TXNIP	[95]
		Breast cancer	Non	Tak1	USP8 stimulates the JNK pathway and stabilizes Tak1 to encourage the migration of breast cancer cells	[96]
		NSCLC	Non	PD-L1	LncRNA SNHG12 promotes USP8 to stabilize PD-L1 by regulating the increase of HuR, thus promoting the growth and immune escape ability of NSCLC	[97]
USP9X		OSCC	Upregulated	MCL-1	USP9X promotes the occurrence of oral cancer by stabilizing MCL-1 and leads to poor prognosis of oral cancer	[98]
		CC	Downregulated	EGLN3	By stabilizing EGLN3, USP9X controls KIF1B in a manner that is anticancer	[99]
USP10		Breast cancer	Non	CircWSB1	By controlling USP10 to maintain the expression of p53, CircWSB1 accelerates the growth of breast cancer	[100]
		HCC	Upregulated	YAP/TAZ	USP10 stabilizes YAP/TAZ and accelerates the development of hepatocellular cancer	[101]
		GBM	Upregulated	RUNX1	By controlling the stability of RUNX1, USP10 preserves the MES features of GBM cells and encourages the growth of GBM	[102]
USP11		Breast cancer	Upregulated	p21	By keeping p21 stable, ERK1/2-phosphorylated USP11 encourages the growth of breast cancer cells	[103]
USP12		MM	Upregulated	HMGB1	USP12 induces MM autophagy and bortezomib resistance by stabilizing HMGB1	[104]
USP13		Cervical cancer	Upregulated	Mcl-1	By keeping Mcl-1 stable, USP13 encourages the growth of cervical cancer cells	[105]
		ccRCC	Upregulated	ZHX2	By stabilizing ZHX2, USP13 contributes to the growth of ccRCC tumors	[106]
USP14		PDAC	Upregulated	TAZ	USP14 promotes the progression of PDAC and liver metastasis by regulating TAZ	[107]
		Colorectal cancer	Upregulated	JNK	USP14 promotes colorectal tumorigenesis by stabilizing JNK protein and further activating MAPK/JNK signaling pathway	[108]
USP15		Lung cancer	Downregulated	BECN1	By controlling TRAF6-BECN1, USP15 causes autophagy and inhibits the growth of lung cancer	[109]
USP16		PCa	Upregulated	c-Myc	USP16 promotes PCa cell proliferation by stabilizing c-Myc	[110]
USP17		ccRCC	Non	SETD8	Renal cell carcinoma tumor progression and metastasis are accelerated by SETD8’s regulation of SREBP1 in the presence of USP17-stabilized SETD8	[111]
USP18		Pancreatic cancer	Upregulated	Notch1	USP18 promotes the progression of pancreatic cancer by stabilizing Notch1 and regulating Notch1-c- myc pathway	[112]
USP19		Breast cancer	Upregulated	Non	In breast cancer, USP19 enhances cancer cell migration and invasion and is a sign of poor prognosis	[113]
USP20		Breast cancer	Upregulated	SNAI2	By keeping SNAI2 stable, USP20 encourages breast cancer metastasis	[114]
USP21		GBM	Upregulated	FOXD1	USP21 promotes the growth and development of GBM by stabilizing FOXD1	[115]
USP22		CCA	Upregulated	SIRT1	USP22-regulated SIRT1 combined with Akt/ERK for deacetylation promotes the malignant growth of CCA in an epigenetic way	[116]
		HCC	Upregulated	ZEB1	By maintaining ZEB1 and boosting VEGFA transcription, USP22 encourages the development and angiogenesis of hepatocellular carcinoma	[117]
		Breast cancer	Upregulated	ERα	By keeping ER stable, USP22 fosters the development and division of breast cancer cells	[118]
USP24		Bladder cancer	Non	GSDMB	By controlling GSDMB and turning on the STAT3 pathway, USP24 promotes the proliferation and invasion of bladder cancer cells	[119]
USP25		PDAC	Upregulated	HIF-1 α	USP25 maintains PDAC cell survival by regulating the stability and transcriptional activity of HIF-1 α	[120]
USP26		ATC	Upregulated	TAZ	USP26 regulates TAZ stability to promote anaplastic thyroid carcinoma	[121]
USP27		HCC	Upregulated	SETD3	By controlling SETD3’s stability, USP27 encourages liver tumor growth and carcinogenesis	[122]
USP28		PC	Upregulated	FOXM1	By stabilizing FOXM1 and triggering the Wnt/β-catenin signaling pathway, USP28 promotes PC growth	[123]
USP29		HCC	Non	HIF1α	USP29 stabilizes HIF1α stability up-regulates sugar degradation to promote HCC resistance to Sorafenib	[124]
USP30		OSCC	Upregulated	c-Myc	USP30 promotes the development of OSCC by regulating c-Myc, and MF-094 plays an anti-tumor role by targeting USP30/c-Myc signal pathway	[125]
USP33		CRPC	Upregulated	DUSP1	By maintaining DUSP1 and preventing JNK activation, USP33 makes prostate cancer cells resistant to docetaxel by preventing apoptosis	[126]
USP34		PC	Upregulated	Non	By blocking the PRR11 and p38 MAPK signaling pathways, down-regulated USP34 restricts the proliferation and migration of PANC-1 cells	[127]
USP35		NSCLC	Non	BIRC3	By controlling the stability of BIRC3, USP35 increases the resistance of non-small cell lung cancer cells to cisplatin	[128]
USP36		ESCC	Upregulated	YAP	By controlling the stability of YAP, USP36 can increase the risk of cancer	[129]
USP37		GC	Non	Snail1	Through the control of Snail1 through USP37, PLAGL2 encourages the migration and proliferation of gastric cancer cells	[130]
USP38		Colorectal cancer	Downregulated	HDAC3	Through the control of HDAC3, USP38 prevents the development of colorectal cancer	[131]
USP39		HCC	Upregulated	ZEB1	By maintaining the ZEB1 protein level, USP39 accelerates the development of hepatocellular carcinoma	[132]
USP41		Breast Cancer	Upregulated	Snail	USP41 Stabilizes Snail to Promote Breast Cancer Progression	[133]
USP43		Breast Cancer	Non	NFAT2	Cav2.2 promotes breast cancer invadopdia formation and metastasis by regulating USP43 through NFAT2	[134]
USP44		NPC	Downregulated	TRIM25	USP44 regulates Ku80 through TRIM25 to enhance DNA damage and inhibit the development of nasopharyngeal carcinoma	[135]
USP45		SOC	Non	Snail	MYH10 and MYH9 promote SOC development and cisplatin resistance by regulating Snail through USP45	[136]
USP46		ESCC	Upregulated	ENO1	By maintaining the stability of ENO1, USP46 encourages the migration and invasion of ESCC cells	[137]
USP47		CRC	Upregulated	YAP	USP47 accelerates the development of colorectal cancer via controlling the stability of YAP	[138]
USP48		HCC	Downregulated	SIRT6	Mettl14 controls the stability of SIRT6 through USP48 to slow the growth of hepatocellular cancer	[139]
USP49		GC	Upregulated	YAP1	USP49 promotes GC progression and chemotherapy resistance by stabilizing YAP1	[140]
USP51		LUAD	Upregulated	ZEB1	A CDK4/6 phosphorylation-activated USP51 stabilizes Human lung adenocarcinoma ZEB1 enhances invasion and metastasis	[141]
USP53		HCC	Downregulated	CYCS	By controlling the stability of CYCS, USP53 prevents hepatocellular carcinoma cells from proliferating, migrating, and invading	[142]
USP54		GC	Non	PLK4	CEP120 regulates the stability of PLK4 through USP54 to promote centrosome amplification and GC progress	[143]
CYLD		NPC	Downregulated	p53/FZR1	CYLD inhibits glycolysis and tumor growth of nasopharyngeal carcinoma by regulating PFKFB3 stability through p53 and FZR1	[144]
		PC	Downregulated	NoxO1	CYLD participates in the progression of prostate cancer by regulating NoxO1 protein	[145]

*USP* ubiquitin hydrolase structural domain, *ZnF* zinc finger ubiquitin-specific protease domain, *DUSP* domain in USPs, *UBL* ubiquitin-like, *UBA* ubiquitin-associated domain, *TBC* the Tre2-Bub2-Cdc16 domain-containing RAB-specific GTPase-activating proteins, *TRAF* TNF-receptor associated factor, *catalytic* catalytic domains, *M ZnF-MYND* MYND (myeloid, nervy and DEAF1)-type zinc fingers, *MIT* microtubule interaction and transport, *Rhod* Rhodanese domain, *Ataxin 2C* Ataxin 2-like carboxy-terminal domain, *NT* N-terminal domain, *CS* CHORD-SGTI domain, *TM* transmembrance, *NES* nuclear export sequence, *iUSP* inactive USP domain, *UIM* ubiquitin interaction motif, *AR* arg rich domain, *PR* proline-rich (PR) motifs, *B* B-box, *SIM* SUMO-interacting motif, *CAP* CAP-Gly domain, *HCC* hepatocellular carcinoma, *PDAC* pancreatic ductal adenocarcinoma, *PC* prostate cancer, *NPC* nasopharyngeal carcinoma, *TNBC* triple-negative breast cancer, *GBM* glioblastoma, *GC* gastric cancer, *ESCC* esophageal squamous cell carcinoma, *GBC* gallbladder cancer, *NSCLC* non-small cell lung cancer, *LUAD* lung adenocarcinoma, *OSCC* oral squamous cell carcinoma, *CC* cholangiocarcinoma, *MM* multiple myeloma, *ccRCC* clear cell renal cell carcinoma, *PCa* prostate cancer, *CCA* cholangiocarcinoma, *ATC* anaplastic thyroid cancer, *CRPC* castration-resistant prostate cancer, *SOC* serous ovarian cancer, *CRC* colorectal cancer.

**Fig. 2:**
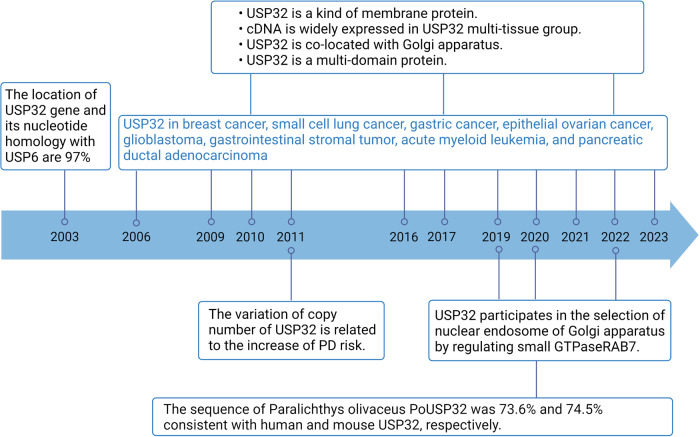
USP32 study schedule. Selected findings are listed and antineoplastic effects are labeled blue. PD: Parkinson’s disease.

The USPs family includes the ubiquitin-specific protease 32 (USP32), also known as NY-REN-60, which codes an ancient and unique gene. USP32 highly conserved homologous genes are evident in all intact post-animal genomes [22]. USP32 is a newly identified de-ubiquitin enzyme in recent years, which was first described in a research article in 2003, and then the related research on USP32 was gradually unveiled (Fig. 2). In recent years, the importance of USP32 continues to be revealed, especially in cancer, where USP32 is often disordered.

## Properties of USP32

### USP32 chromosomal location, subcellular localization

**Fig. 3:**
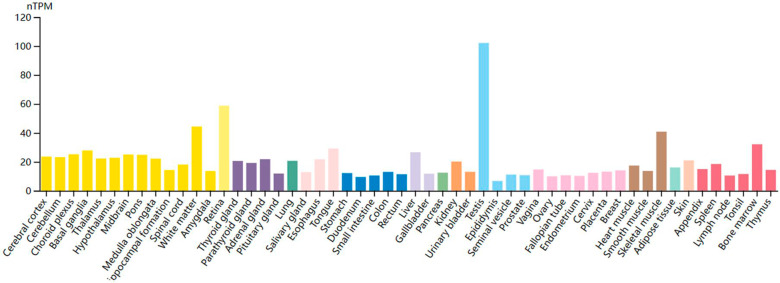
USP32 RNA in various tissues of human body. Available online: https://www.proteinatlas.org/ENSG00000170832-USP32/tissue (accessed on 26 March 2023).

The ancient and highly conserved human USP32 gene is found on the chromosomal band 17q23 [22]. According to the findings of the multi-tissue cDNA panel, USP32 was highly expressed in the leukocytes, thymus, spleen, testis, prostate, ovary, small intestine, and colon [23]. We queried USP32 in human tissues through the Human Protein Atlas website, the testis showed the greatest RNA expression of USP32 (Fig. 3). USP32 is upregulated in a variety of cancers, including small cell lung cancer [24], gastric cancer [25, 26], breast cancer [23, 27, 28], epithelial ovarian cancer [29], glioblastoma [30], gastrointestinal stromal tumor [31], pancreatic duct adenocarcinoma [32] and acute myeloid leukemia [33].

Endogenous USP32 is found in the cytoplasm and membrane, according to the findings of a subcellular separation experiment [34] and a fluorescence protection experiment [23]. This is consistent with earlier findings that USP32 is an active membrane-bound ubiquitin protease [24]. In the investigation of drug resistance in tumor cells, USP32, a membrane protein, can result in resistance to the anticancer medication YM155 by interfering with the steady expression of SLC35F2 [28]. Subcellular localization studies also show that USP32 may be co-located with Golgi [23], and some studies have found that USP32 can regulate the participation of small GTPase Rab7 in Golgi endosome selection [35]. The absence of USP32 will also affect the structure and function of intracellular lysosomal vesicles and the transport of nuclear endosomes, thus affecting the occurrence of some diseases [35].

### The structure and activities of USP32

**Fig. 4:**
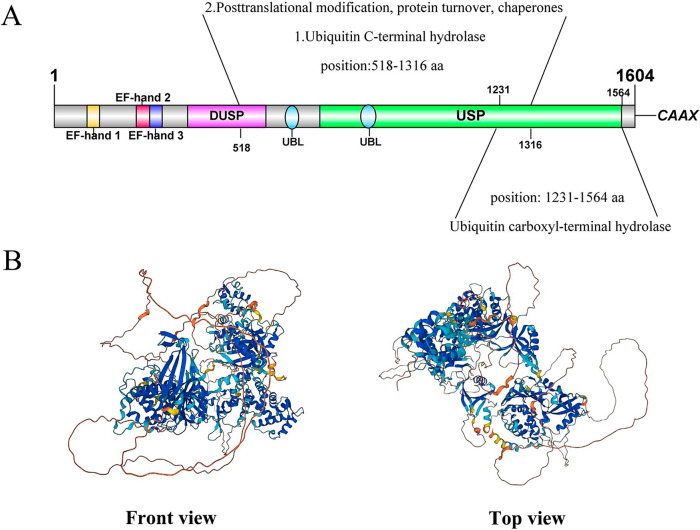
Schematic diagram of USP32 structure. **A** schematic diagram of the composition of the USP32 domain; **B** the front and top view directions of the USP32 structure. Available online: https://alphafold.ebi.ac.uk/entry/Q8NFA0 (accessed on March 25th, 2023).

Human USP32 is composed of 1604 amino acids [23] and has an estimated molecular weight of 182KDa. The multi-domain protein includes calcium-binding EF-hand with signal transduction mechanism, DUSP (domain in USP) expected to mediate protein-protein interaction, a USP catalytic domain, and two ubiquitin-like (UBL) domains and c-terminal prenylation sites (CAAX box) (Fig. 4A) [36]. Among them, the USP catalytic structural domain is present in all member structures of the USPs and is the most important functional structural domain in the USPs [37], containing key cysteine and histidine residues [38]. The USP catalytic domain has strong homology in the regions around catalytic Cys box and His box [37]. All human DUBs have the two distinct structures of the N-terminal calcium-binding EF hand and the C-terminal prenylation site (CAAX box), the latter of which is present only in the USP32 structure and is associated with USP32-doped membrane structures [34]. Researchers discovered that the USP domain in USP32, which has substrates for both mono- and double-ubiquitin cleavage, does not often tend to break one of the eight ubiquitin connections, M1, K6, K11, K27, K29, K33, K48, and K63 [35]. In addition, a ubiquitin C-terminal hydrolase (UBP12) exists in the 518-1316 amino acid position of the USP32 structure, which can undergo post-translational modification and protein turnover, while the ubiquitin carboxyl terminal hydrolase has the 1231-1564 amino acid position of the USP32 structure (Fig. 4A). The structure of USP32 predicted by Alpha Fold [39, 40] is shown in Fig. 4B.

USP32 has a variety of molecular functions and characteristics. Firstly, USP32 is widely expressed in tissues and has the conservative peptidase characteristics of ubiquitin-specific proteases [23]. USP32 locates on the cell membrane by lipid anchoring and catalyzes the conversion of C-terminal thioesters to free ubiquitin and mercaptan, which may affect a variety of cell processes. Secondly, USP32 supports the role of Rab7 in the transport and circulation of MVB through two different mechanisms [35]. In a genome-wide sense, USP32 is linked to a higher incidence of Parkinson’s disease [41]. USP32 may also be the target gene of some miRNAs and participates in the ubiquitin proteolysis pathway [42]. USP32 gene also has sulfhydryl-dependent ubiquitin-specific protease activity, which can bind calcium ions [43]. The ability of USP32 to bind to proteins or protein complexes allows it to take part in the Golgi’s nuclear endosome selection process [44]. As a result, USP32 is engaged in a variety of biological activities. The following sections will explain USP32’s specific roles and mechanisms.

### A homologue of USP32

#### USP32 and USP6

In human samples, the homologue of USP32 is USP6 (also known as TRE2). In actuality, phylogenetic study reveals that it is a chimera of two genes, USP32 (NY-REN-60) and TBC1D3, and shares 97% of its nucleotides with USP32 [22]. More than 20 years ago, USP6 was discovered to be a new oncogene [45]. It functions as a cysteine protease to control the degradation of ubiquitin and to take part in a number of cellular functions, such as intracellular transport, protein synthesis, inflammatory signaling, and cell transformation [46]. USP6 is a gene chaperone that has been discovered to have numerous partners. When these partners fuse with USP6, USP6 is transcriptionally activated and participates in the Wnt/β-catenin pathway, NF-κB pathway, and JAK1-STAT3 pathway, among other pathways involved in tumorigenesis [47]. Meanwhile, both USP6 and USP32 as ubiquitin protein hydrolases have conserved enzyme structural domains [22].

#### USP32 and PoUSP32

We know that USP32 is widely expressed in mammals and is involved in tumor activity, fragile X syndrome [48] and chronic nephropathy [42]. There is no previous record of USP32 in fish, and a recent study found that the sequence of Japanese flounder PoUSP32 is 73.6% and 74.5% identical to that of human and mouse USP32, respectively. The PoUSP32 of Japanese flounder consists of 1613 amino acid residues, which is expressed in 9 different tissues of Japanese flounder, but the expression level is significant in the intestine. PoUSP32 is similar to mammalian USP32 in that it has the conserved enzyme domain of USPs family and the conservative function of deubiquitin. In order to better understand the role of PoUSP32, scientists discovered through in vivo and in vitro investigations that pol-miR-363-3p48 interacts with the 3’UTR of PoUSP32 and decreases the expression of PoUSP32. Vitro experiments prevented dolphin infection with Streptococcus by down-regulation of PoUSP32 or overexpression of polmiR-363-3p in toothfish cells. Vivo research have shown that overexpression of USP32 and interfering with pol-miR-363-3p can increase Streptococcus dolphin infection in Paralichthys olivaceus tissue. These findings imply that pathogen infection substantially affects the regulation of PoUSP32 and pol-miR-363-3p expression [49].

## The role of USP32 in several cancers

**Fig. 5:**
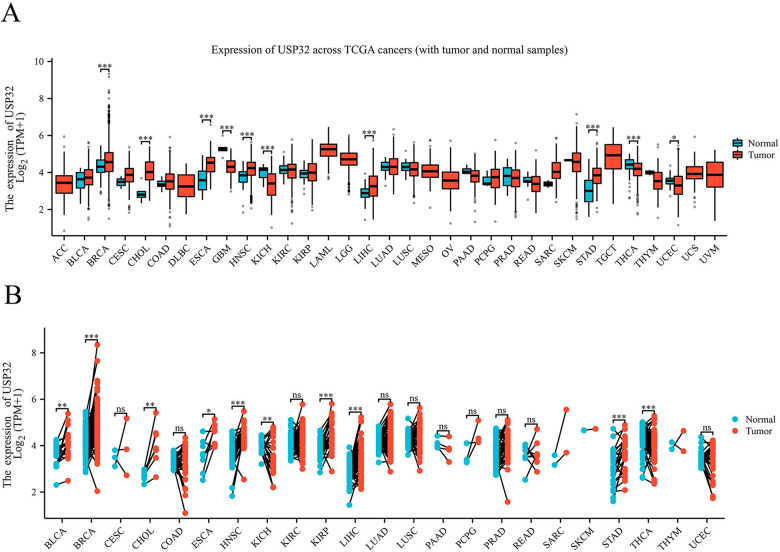
TCGA data on USP32 expression in various malignancies. **A** The level of USP32 mRNA expression in tumor tissues and surrounding tissues according to the TCGA database. **B** USP32 expression levels in nearby normal tissues and matched malignant tissues from human cancer patients, according to the TCGA database. Box plots showing USP32 log2(TPM) expression in various malignancies and log2(TPM) transcript count per million. USP32 expression is denoted by the colors red and blue in normal and malignant tissues, respectively. The Wilcoxon test was used to determine the significant difference in USP32 expression between tumor and normal tissue. The significance level is indicated by the number of stars on top of the box plots (*p < 0.05, **p < 0.01, ***p < 0.001).

In recent years, it has been found that USP32 can regulate the growth and development of different tumors through the catalytic activity of deubiquitinating enzymes. We investigated the expression of USP32 in various malignant tumors by using the TCGA database and assessed the expression of USP32 in 33 tumor tissues and paraneoplastic tissues (Fig. 5). In terms of statistical significance, six of them, including cholangiocarcinoma (CHOL), esophageal carcinoma (ESCA), head and neck squamous cell carcinoma (HNSC), hepatocellular carcinoma (LIHC), and stomach adenocarcinoma (STAD), displayed increased levels of USP32 mRNA expression. However, in four additional tumor samples-glioblastoma multiforme (GBM), kidney suspicious cell (KICH), thyroid cancer (THCA), and endometrial cancer (UCEC)-USP32 gene expression were dramatically downregulated (Fig. 5A). Unexpectedly, glioblastoma has a low expression level of USP32 in the TCGA database. This outcome does not appear to be in line with those mentioned in the literature. We posit that this finding may be explained by post-translational alteration, tumor heterogeneity, or a small sample size. Thus, we need to learn more about USP32 expression in glioblastoma and its function. Additionally, we examined TCGA data sets to find USP32 expression in human cancer tissues that were linked with nearby normal tissues. We found that compared to normal tissue, USP32 expression was significantly elevated in 10 of 23 cancers, such as bladder thelial carcinoma (BLCA) and breast invasive carcinoma (BRCA), cholangiocarcinoma (CHOL), esophageal carcinoma (ESCA), head and neck squamous cell carcinoma (HNSC), kidney chromophobe (KICH), kidney renal papillary cell carcinoma (KIRP), liver hepatocellular carcinoma (LIHC), stomach adenocarcinoma (STAD) and thyroid carcinoma (THCA) (Fig. 5B). The increased expression of USP32 in different cancers is a necessary condition for cell proliferation and tumorigenesis. According to several studies, high levels of USP32 expression are associated with poor prognosis in various malignant tumors. In order to fully understand the mechanism of action of USP32 in different malignant tumors, researchers need to learn USP32 and its regulation of downstream targets.

### SCLC

**Fig. 6:**
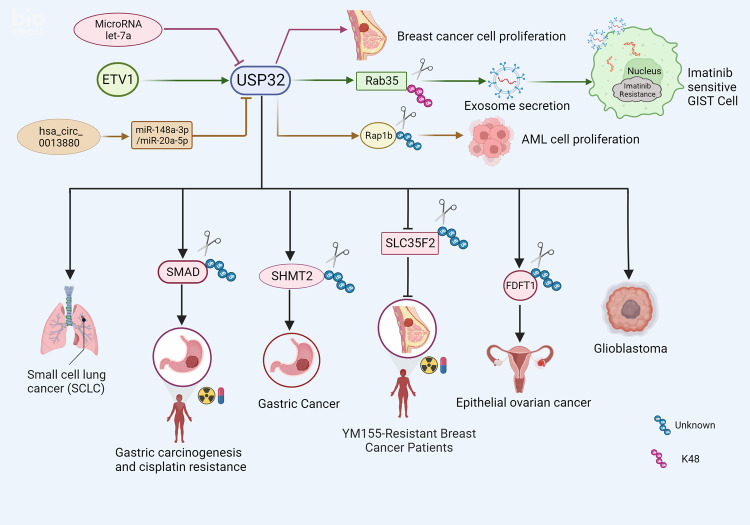
USP32 the function of many malignancies. The expression of USP32 protein is negatively correlated with MicroRNAlet-7a in breast cancer, which can suppress USP32 expression and impede the growth of breast cancer cells. ETV1 controls the expression of USP32, which in turn stabilizes Rab35 by ubiquitinating Lys48 (K48) on Rab35 and increases exocrine secretion of imatinib-resistant gastrointestinal stromal tumors. USP32 supports the growth of acute myeloid leukemia (AML) through deubiquitinating and stabilizing Rap1b, while hsa_circ_0013880 controls USP32 expression in AML through miR-148a3p/miR-20a-5p. USP32 is extensively expressed in lung cancer tissues, where it dramatically speeds up cell division, encourages cell growth, and prevents cell death. USP32 contributes in the onset and progression of gastric cancer by controlling SHMT2, and USP32 enhances gastric carcinogenesis and cisplatin resistance in gastric cancer by eliminating ubiquitin and stabilizing SMAD2. USP32 increases resistance to YM155 in breast cancer cells through modulation of SLC35F2. By controlling the expression of FDFT1 protein, USP32 contributes to the development and progression of epithelial ovarian cancer. By controlling the cell cycle, DNA replication, basal excision repair, and mismatch repair in glioblastoma, USP32 promotes GBM.

About 15% of all lung cancers are small cell lung cancers (SCLC), which are distinguished by an unusually high rate of proliferation, significant early metastasis, and a poor prognosis [50, 51]. The preferred first- and second-line treatment for small cell lung cancer (SCLC) remains chemotherapy [52]. For small-cell lung cancer, USP32 is thought to be a potential therapeutic target. According to a study, USP32 is highly expressed in small-cell lung cancer. The association between high levels of USP32 and low survival rates was significant. In vitro experiments confirm that USP32 promotes tumor growth. It is interesting to note that USP32 can influence the development of small-cell lung cancer’s cell cycle. USP32 knockdown increased apoptosis in addition to causing cell cycle arrest in the G0/G1 phase, with concomitant increases in Caspase3, cleaved PARP and P53-related apoptotic proteins. The marker protein of epithelial–mesenchymal transformation (EMT) may be affected by the deletion of the USP32 gene, which can upregulate the expression of E-calcineurin and down-regulate the expression of N-calcineurin protein. Thus, the ability of small cell lung cancer cells to proliferate and generate clones, as well as the ability of cells to invade and migrate, could be inhibited by interfering with USP32 [24] (Fig. 6). These results will help to elucidate how small cell lung cancer tumors progress and guide the establishment of therapeutic targets for small cell lung cancer.

### Gastric cancer

Gastric cancer is characterized by high incidence, poor prognosis, and cellular and molecular heterogeneity, which makes it a major health problem in the world [53]. Effective treatments for gastric adenocarcinoma include systemic chemotherapy, radiation, surgery, immunotherapy, and targeted therapy [54]. Each type of gastric cancer has its own characteristics, and each gastric cancer patient can be effectively treated clinically through molecular targeting therapy [55]. Researchers have found a link between SMAD2 and USP32. SMAD2 is a key element of transforming growth factor signaling pathway in the occurrence and development of gastric cancer. USP32 has been discovered as an oncogene implicated in the development and metastasis of GC cells due to the considerable inhibition of GC cell proliferation and migration caused by USP32 gene knockout or deletion in both vivo and in vitro. It is more likely that USP32 will be used as a possible biomarker and therapeutic target for gastric cancer because of the high expression of USP32 in this disease and its relationship to the short overall survival rate and high T stage of gastric cancer patients. The scientists discovered that USP32 modulates SMAD2 expression in a ubiquitin protease-dependent manner. Gastric cancer cells’ cisplatin resistance can be decreased by down-regulating USP32 expression, indicating that USP32 is involved in the development of cisplatin resistance. Meanwhile, rescue experiments showed that overexpression of SMAD2 could reverse the effects of silenced USP32 on gastric cancer cell growth, metastasis and cisplatin resistance. These observations suggest that SMAD2 is located downstream of USP32 in GC, and USP32 could regulate the expression of SMAD2 and promote gastric carcinogenesis and cisplatin resistance by enhancing the stability of SMAD2 protein in gastric cancer cells, so targeting USP32 may be a potential therapeutic strategy for GC [25] (Fig. 6).

Another study found that early and late stages of gastric cancer had higher expression levels of the USP32 and SHMT2 proteins, and immunohistochemistry analysis supported these findings. The researchers also found that after the silencing of USP32 expression in gastric cancer, the expression of SHMT2 decreased [26] (Fig. 6). Therefore, targeting the USP32-SHMT2 axis may provide some ideas for the treatment of gastric cancer.

### Breast cancer

According to the most recent report on cancer statistics, breast cancer continues to be the most common tumor among women [56]. Because the occurrence and development of tumors are regulated by complex molecular mechanisms, the choice of treatment options and disease prognosis of breast cancer are challenged at the molecular level [57]. USP32 was found to have an elevated copy number in estrogen receptor (ER) positive tumors, which is one of the 81 gene copy number traits that predict the metastatic capacity of breast cancer [58]. Meanwhile, it has been noted that USP32 is one of the transcripts that are up-regulated in malignant breast epithelial cells [59], indicating that USP32 may be a helpful biomarker in a subclass of breast cancer cells. In one work, USP32 transcript analysis was expanded to breast cancer cell lines and primary tumors in order to examine USP32 expression in breast cancer cells. USP32 was discovered to be overexpressed in both primary breast tumors and breast cancer cell lines. MCF7 cells, a typical cell line in which USP32 was overexpressed, were examined for mutations, but none were found, demonstrating that USP32 was overexpressed in wild-type transcripts [23] (Fig. 6).

In another study, we found that microRNA let-7a reduced the amount of USP32 protein in MCF-7 cells. USP32 was identified by bioinformatics study as a miR let-7a target gene. The 3’UTR location of USP32 mRNA was found to be the target of miR let-7a, and miR let-7a was able to reverse the reduction in ubiquitin levels caused by USP32. As a result, miR let-7a might suppress the expression of USP32 protein by directly binding to the USP32 3’UTR. As a result, miR let-7a can regulate USP32 in BCa, which can decrease proliferation and offer a new potential target for the treatment of breast cancer [27] (Fig. 6).

USP32 has reportedly been linked to the medication resistance mechanism in breast cancer. The uptake of the anti-cancer drug candidate YM155 by breast cancer cells is controlled by the expression of a solute carrier protein called SLC35F2. In contrast to SLC35F2, USP32 is highly expressed in breast, colon and lung malignancies. These cancer cells are resistant to YM155, a small molecule drug that targets a variety of cancers. Researchers further found that the direct binding of USP32 and SLC35F2 in the endoplasmic reticulum could negatively regulate the stability of SLC35F2 and promote drug resistance to YM155 in breast cancer cells [32] (Fig. 6). Therefore, inhibition of USP32 can increase the protein expression of SLC35F2 and improve the chemotherapeutic effect of YM155 on breast cancer, opening up a new pathway for clinical drug resistance treatment.

### Epithelial ovarian cancer

The most frequent type of gynecological cancer to cause death is epithelial ovarian cancer, which has a high mortality rate in part because of the patient’s late stage at the time of diagnosis [60]. The focus of immunotherapy in EOC clinical trials is similar to those of many other cancer subtypes, and programmed cell death 1 (PD-1) is its main inhibitor [61]. The standard of care for all female patients with epithelial ovarian cancer is the genetic identification of altered genes that affect treatment [62]. In one study, the researchers used the pertinent database search to discover that USP32 was substantially expressed in human ovarian cancer peritoneal tumors and was regulated in ovarian cancer. Epithelial ovarian cancer has a bad prognosis, which is directly associated with USP32. A peritoneal tumor’s ability to metastasize can be prevented by inhibiting USP32. Farnesyl-diphosphate farnesyltransferase 1 (FDFT1) in the valproic acid pathway is a new USP32 substrate regulated by the UPS, according to proteomic research. FDFT1, an oncogene and suppressor gene, has a direct connection to the growth of stem cells and cancerous cells. They showed that USP32 and FDFT1 expression was higher in tumor spheroids than in adnexal cells, and that the FDFT1 inhibitor ZA, blocking USP32, or interfering with the mevalonate pathway might inhibit the formation of tumor spheres in epithelial ovarian cancer (Fig. 6). These results suggest that USP32-FDFT1 axis promotes the migration of epithelial ovarian cancer and may become a new target for EOC therapy [29].

### Glioblastoma

The most prevalent and aggressive high-grade primary malignant tumor of the adult central nervous system (CNS), glioblastoma has a very bad prognosis [63, 64]. Combination therapy may yield better results because the survival rate of currently approved GBM treatments is low and immunotherapy for GBM typically lacks sufficient clinical expertise to translate into meaningful benefits [65]. The elevated expression of USP32 protein in GBM tissues was validated by a study. Glioblastoma cell lines (Umur118 MG, Umur87 MG, A172, T98G, and Umur251 MG) had higher mRNA and protein levels of USP32 than normal brain cells SVG p12 [30]. In addition, they found that the higher the expression of USP32 in GBM, the worse the prognosis. Cancer cell proliferation and migration can be stopped in vitro by inhibiting USP32, and tumor growth can be stopped in vivo by down-regulating the USP32 gene. In their research, they discovered that USP32 can speed up the transition of the cell cycle from the G0 phase to the G1 phase [30], start DNA replication, and so enhance the growth of cancer cells. Then, RT-qPCR tests were used to confirm the effects of USP32 on a few functional molecules. The findings demonstrate that USP32 controls several genes’ expression and is a key oncogene in basal excision repair and mismatch repair [30] (Fig. 6). Therefore, USP32 is a promising new molecular target, from which researchers can study new treatments to control the development of glioblastoma.

### Gastrointestinal stromal tumor

The gastrointestinal stromal tumor is a kind of malignant stromal tumor, which is treated separately because of its special histogenesis, clinical manifestation, and specific treatment [62]. Local GIST can be treated by surgery [66], while imatinib can be used for unresectable advanced and metastatic gastrointestinal stromal tumors [67]. However, about half of the patients will develop secondary imatinib resistance after two years [68]. Recent research has revealed that the USP32-Rab35 axis is crucial for controlling treatment resistance in gastrointestinal stromal tumors. The exocrine secreted by tumor cells has a great influence on the transmission of drug resistance in drug-sensitive cells. Previous studies have found that Rab35 regulates exocrine secretion through exosome recovery and early sorting [69]. A recent study discovered and concluded that Rab35 can influence the spread of drug resistance by controlling the exocrine secretion of gastrointestinal stromal tumors, and that gastrointestinal stromal tumors considerably overexpressed Rab35 [31]. In terms of mechanism, the expression of USP32 and transcription factor ETV1 have a positive correlation. In gastrointestinal stromal tumors that are imatinib-resistant, ETV1 increases the expression of USP32. USP32 promotes the exocrine secretion of imatinib-resistant gastrointestinal stromal tumors by decreasing the Lys48 (K48) ubiquitination of Rab35, protecting it from proteasome destruction (Fig. 6). Their research suggests that individuals with gastrointestinal stromal tumors resistant to imatinib may benefit from focusing on the USP32-Rab35 axis as a potential therapeutic target.

### Acute myeloid leukemia

A lethal myeloid malignant tumor that develops in the blood system is acute myeloid leukemia (AML) [70]. According to WHO, the tumor is divided into four categories. It is mainly characterized by primordial and juvenile myeloid cells in the bone marrow and peripheral blood, but does not include lymphoid cell dysplasia, accompanied by infection, anemia and bleeding symptoms [71]. The disease can occur at any age, but mainly in the elderly [72]. At present, there are many options for the treatment of AML. The realization of complete response (CR) is the general method for the treatment of AML. Other treatment methods include induction therapy, induction therapy after remission, non-intensive therapy for newly diagnosed patients, recurrent refractory AML, etc [73]. A study reveals the regulatory mechanisms of USP32 and circRNAs in acute myeloid leukemia. In AML patients, the expression of USP32 and hsa_circ_0013880 is up-regulated and positively linked. Hsa_circ_0013880 overexpression can either positively regulate USP32 protein and increase AML cell proliferation. Through the analysis of bioinformatics and related experiments, we know that miR-148a3p/miR-20a-5p can regulate USP32, and hsa_circ_0013880 can combine with miR-148a3p/miR-20a-5p. In the BMNCs of patients with AML, the researchers found that USP32 acts as a well-intentioned deubiquitinase for Ras-related proteins (Rap1b). USP32 could interact with Rap1b and stabilize Rap1b. Knockdown of USP32 in HL-60 and U937 cells significantly reduced the expression of Rap1b. Overexpression of Rap1b largely reversed USP32 knockdown-induced apoptosis. As a result, the hsa_circ_0013880/USP32/-Rap1b axis plays a crucial regulatory role in the emergence and progression of AML (Fig. 6). Future studies of this axis may provide a new approach to the treatment of acute myeloid leukemia [33].

## Conclusions

There are two main ways of protein degradation in human cells: one is in lysosome, which mainly degrades foreign proteins and has poor selectivity for proteins. The other is degraded in the proteasome, via an ATP-dependent pathway (energy-demanding), after ubiquitination modifications, which mainly degrades intracellular proteins with abnormal structure and short life span. The polyubiquitination of substrate proteins and proteasome degradation can affect or regulate a wide range of physiological processes, including gene transcription, cell cycle regulation, immune response, cell receptor function, tumor growth, inflammatory process, and others. As a member of the USPs family, USP32 controls DNA damage repair, cell cycle, cancer-related signaling, and protein stability.

This study analyzes the current findings on USP32, including its structure and biological functions, and discusses how it regulates the growth of various types of tumors. The presence of endogenous USP32 in both the cell membrane and cytoplasm determines that USP32 can be involved in the regulation of a number of small molecules. Meanwhile, USP32 is a multi-structural domain protein that possesses multiple protein properties and biological functions. Current researchers have found that the homologs of USP32 in human samples and in fish are USP6 and PoUSP32, respectively, which share the conserved enzyme structural domains of the USPs family and deubiquitination functions. USP32 is expressed in a wide range of tissues, but its expression is frequently dysregulated in some tumors. Therefore, a possible target for cancer therapy is USP32, which has been shown an important role in many malignant tumors and chemotherapy resistance. In light of the fact that USP32 is a target for the treatment of tumors and is dependent on ubiquitin-modified target genes as oncogenes, more research on USP32 as a means of regulating the efficacy of cisplatin and other DNA-damaging drugs should be done. This necessitates a comprehensive investigation, review, and evaluation of its therapeutic significance and application.
